# Evaluation of the Corrosion Resistance Properties of Electroplated Chitosan-Zn_1−x_Cu_x_O Composite Thin Films

**DOI:** 10.3390/nano7120432

**Published:** 2017-12-06

**Authors:** Anandhavelu Sanmugam, Dhanasekaran Vikraman, K. Karuppasamy, Ji Young Lee, Hyun-Seok Kim

**Affiliations:** 1 Department of Chemistry (S & H), Vel Tech Multitech Dr.Rangarajan Dr.Sakunthala Engineering College, Chennai 600 062, India; sranand2204@gmail.com; 2 Division of Electronics and Electrical Engineering, Dongguk University-Seoul, Seoul 04620, Korea; v.j.dhanasekaran@gmail.com (D.V.); karuppasamyiitb@gmail.com (K.K.); ljy010425@naver.com (J.Y.L.)

**Keywords:** electrochemical, composite thin films, corrosion, morphology, impedance

## Abstract

Novel chitosan–zinc copper oxide (Zn_1−x_Cu_x_O) composites were electrochemically synthesized through galvanostatic deposition. The prepared chitosan-based composite thin films were elaborately investigated to determine their structural, morphological, compositional, impedance, and corrosion properties. X-ray diffraction analysis was performed to reveal their structural orientation of composite thin films. Energy dispersive analysis by X-ray evidently confirmed the existence of Zn, Cu, and O in the composite thin films. Nyquist plots revealed that the chitosan-Zn_1−x_Cu_x_O thin films had obvious semi-circular boundaries, and higher resistance was observed for chitosan-ZnO due to the grain boundary effect. Corrosion properties were evaluated using both an electrochemical method and the ASTM weight gain method, which revealed good corrosion rates of 34 and 35 × 10^−3^ mm/y, respectively, for chitosan-ZnO thin film.

## 1. Introduction

Organic-inorganic nanocomposite materials are attracting substantial attention because of their combination feasibility in the properties of organic and inorganic components [1,2,3,4,5]. Significant interest has been spawned in fabrication of nanocomposite films containing metal oxide nanoparticles in a polymer matrix through electrochemical routes [6,7]. Compared to other preparation routes such as layer-by-layer self-assembly, electrodeposition imparts benefits such as higher deposition rate, shorter processing time, and the possibility of depositing thicker films. Moreover, shape selective fabrication over a uniform film surface with controlled composition can be obtained on different forms of the conducting substrate [8,9]. The fabrication of composite films can be achieved by electrochemical co-deposition of organic and inorganic components [10]. Recent reports have revealed the electrochemical preparation of various natural biomacromolecules such as chitosan [11,12], alginic acid [13], and hyaluronic acid [14,15], and that electrodeposition is a feasible route to fabricate thin and porous structured films [16,17]. Chitosan is an important natural polymer for various applications including biomedical sensors, implants, and anti-microbial and microfluidic devices [12,18,19,20]. Nanocomposite materials based on chitosan with metal oxide materials have become attractive in recent years due to their synergistic behavior [21]. Recently, Sanmugam et al. [21] successfully demonstrated the solvent free synthesis of chitosan-zinc oxide (ZnO) nanocomposites using chitosan and ZnCl_2_. Earlier, Li et al. [22] reported the synthesis of chitosan-ZnO thin films using an electrochemical route. 

ZnO is a semiconductor material with a wide range of applications including biosensors, catalysts, and photovoltaic devices [23,24,25,26,27]. Various methods have been used to synthesize ZnO nanoparticles such as sol-gel, radio-frequency (RF) sputtering, chemical vapor deposition, pulsed laser deposition, and spray pyrolysis [28,29,30,31,32]. In addition, ZnO lattices have be shown to contain various types of defect helping them to behave as n-type semi-conductors due to zinc and oxygen vacancies and interstitials, as well as more complex defects [33,34,35,36,37,38]. Some research groups have modified ZnO properties through adding dopant materials such as N, Al, Ga, Cu, Mg, and In, which can significantly enhance the electrical, dielectric, and optical properties of ZnO [30,32,38,39,40]. Copper oxide (CuO) is a p-type semiconductor material used in a broad range of applications including anti-corrosion properties, solar cells, biosensors, gas sensor, superconductor, lithium-ion electrode materials, magnetic storage media, and field effect transistors [41,42,43,44], and its composite materials have recently attracted attention due to promising features in electrochemical behavior [45,46]. Recently, Arena et al. [47] demonstrated electrochemically-derived chitosan-copper oxide nanocomposites for successful non-enzymatic sensing of hydrogen peroxide.

Corrosion is a common problem which significantly affects the properties of materials [48,49], and high resistance to corrosion is attributed to the spontaneous development of a chemically stable oxide film surface [49,50,51]. Transition metal oxides and conducting polymers are the promising candidates due to their strong bonding structure which can be withstanding in diverse applications including anti-corrosion applications [20,52,53,54,55]. From the detailed investigations of earlier literatures, we have prepared chitosan-zinc copper oxide (chitosan-Zn_1−x_Cu_x_O) composite thin films with various concentrations of zinc and copper chloride by electrosynthesis. To the best of our knowledge, there are no reports available on the electrosynthesis of chitosan-Zn_1−x_Cu_x_O composite thin films. The structural, compositional, morphological, and electrochemical properties of the prepared composite thin films were studied in detail. Hence, we explored the corrosion behavior of electrosynthesized chitosan-Zn_1−x_Cu_x_O composite thin films using an electrochemical and the American standard test method (ASTM) weight loss methods efficiently. The best corrosion resistance performance was exhibited in the chitosan-ZnO thin film, compared with other composite thin films, with the corrosion rates of 34 and 35 × 10^−3^ mm/y by an electrochemical method and the ASTM weight gain method, respectively.

## 2. Results and Discussion

**Figure 1:**
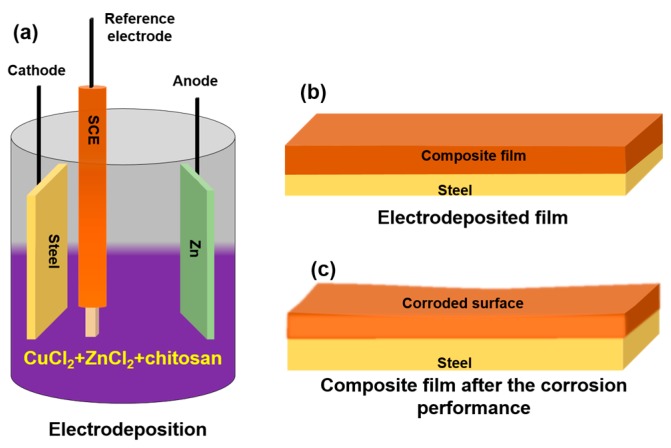
Schematic diagrams of the chitosan-Zn_1−x_Cu_x_O composite thin film. Electrosynthesis (**a**) and structure of the films before (**b**) and after (**c**) corrosion performance testing.

The chitosan-Zn_1−x_Cu_x_O composite thin films were electrochemically synthesized on a mild steel substrate using galvanostatic mode. In order to adjust the Zn and Cu element composition, zinc chloride (ZnCl_2_) and copper chloride (CuCl_2_) concentrations were adjusted in an electrolyte bath. Figure 1a–c illustrates the electrochemically synthesized chitosan-Zn_1−x_Cu_x_O composite thin films on a mild steel substrate, the prepared film structure, and the corroded surface of a composite film post testing, respectively. 

**Figure 2:**
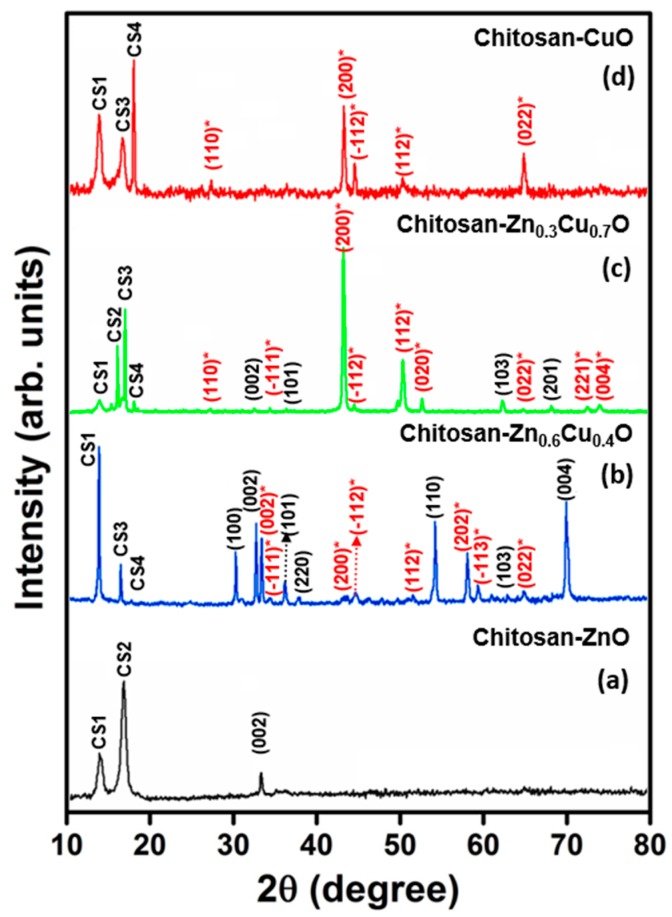
XRD patterns of electrosynthesized composite thin films: (**a**) chitosan-ZnO; (**b**) chitosan-Zn_0.6_Cu_0.4_O; (**c**) chitosan-Zn_0.3_Cu_0.7_O; and (**d**) chitosan-CuO (red color with * indexed peaks are CuO-based lattice planes, black color indexed peaks are ZnO-based lattice planes, and chitosan-based peaks are indexed with prefix CS).

Their structural properties were studied using X-ray diffraction (XRD) analyses. Figure 2a–d shows typical X-ray diffraction patterns of chitosan-ZnO, chitosan-Zn_0.6_Cu_0.4_O, chitosan-Zn_0.3_Cu_0.7_O, and chitosan-CuO composite thin films, respectively. The observed XRD patterns were indexed with joint committee of powder diffraction standard (JCPDS) patterns of CuO (#89-5898 & #78-2076) and ZnO (#89-0511). The XRD pattern in Figure 2a reveals that the electrosynthesized chitosan-ZnO composite thin film exhibited a polycrystalline hexagonal structure. In this XRD pattern, chitosan-related conventional diffraction lines CS1 (13.2°), and CS3 (17.3°) were observed, as has previously been reported [22]. In addition to this, a conventional ZnO (002) lattice orientation peak was present at 2θ = 33.9°, which revealed that chitosan was more dominant than ZnO in the chitosan-ZnO complex matrix. In the XRD pattern of chitosan-Zn_0.6_Cu_0.4_O composite thin film (Figure 2b), strong chitosan-related CS1 (13.1°) and CS3 (17.3°) peaks as well as a low intensity CS4 peak (18.8°) were observed with CS1 being the predominant peak orientation. Moreover, ZnO lattice planes (100), (002), (101), (220), (110), (103), and (004) along with CuO lattice planes (002), (−111), (200), (−112), (112), (202), (−113), and (022), and CuO-related lattice planes (110), (−111), (−112), (112), (020), (022), (221), and (004) with (200) as the predominant orientation were present in the XRD pattern. The ZnO related (002), (101), (103), and (201) lattice orientations were observed for the chitosan-Zn_0.3_Cu_0.7_O thin film (Figure 2c). In addition, conventional chitosan-based CS1 (@13.2°), CS2 (@15.9°), CS3 (@17.3°), and CS4 (@18.7°) peaks were present, which clearly infers that the Zn_1−x_Cu_x_O alloy was fully incorporated into the chitosan polymer. From the XRD pattern of chitosan-CuO composite thin film (Figure 2d), (110), (200), (−112), (112), and (022) lattice planes were predicted for CuO in addition to the chitosan conventional peaks. 

The average crystallite size of chitosan-Zn_1−x_Cu_x_O composite thin films were calculated using the Debye–Scherrer’s equation [56]:

*D* = *K*λ/βcosθ, (1)

where *D* is the crystallite size, *K* is the Scherrer constant, λ is the X-ray wavelength, β is the full-width at half-maximum, and θ is the diffraction angle. The crystallite size was found to be 25, 31, 41, and 38 nm for chitosan-ZnO, chitosan-Zn_0.6_Cu_0.4_O, chitosan-Zn_0.3_Cu_0.7_O, and chitosan-CuO composite thin films, respectively, revealing a decrement in crystallite size in the absence of Zn in the chitosan-CuO composite. 

**Figure 3:**
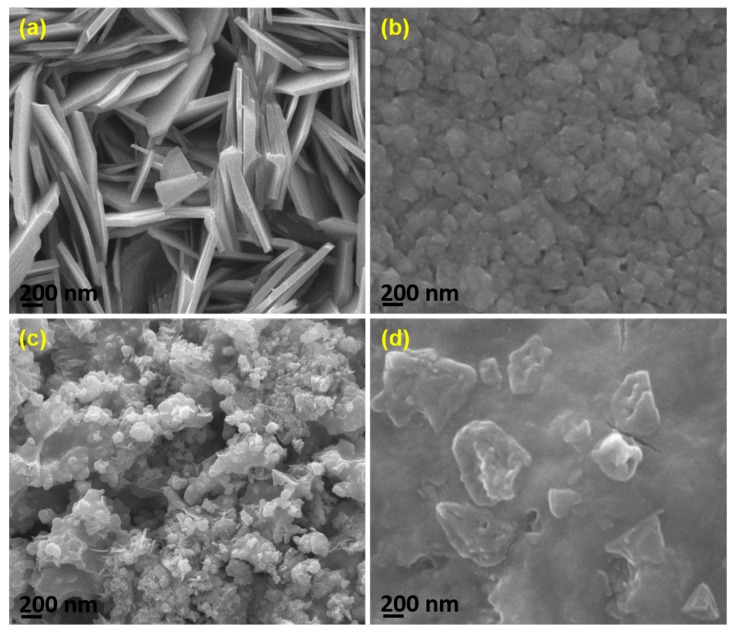
SEM Micrographs of electrosynthesized composite thin films (**a**) chitosan-ZnO; (**b**) chitosan-Zn_0.6_Cu_0.4_O; (**c**) chitosan-Zn_0.3_Cu_0.7_O; and (**d**) chitosan-CuO.

The morphological properties of the composites were studied using scanning electron microscopy (SEM), which was a convenient method for studying the films’ surfaces. The micrographs revealed morphological differences with various combinations of precursor solution with chitosan. Nano-slab-like morphology was observed in the chitosan-ZnO composite thin film, as shown in Figure 3a, in which some discontinuities and overlapping were evident. An inhomogeneous surface with voids and hillocks was observed in the chitosan-Zn_0.6_Cu_0.4_O composite thin film SEM image (Figure 3b). The surface image of chitosan-Zn_0.3_Cu_0.7_O composite thin film is presented in Figure 3c, in which spherical-shaped fine grains covered the entire surface of the film, resulting in smooth surface morphology from the lower grain size. Smooth and uniform surface morphological properties are evident on the surface of the chitosan-CuO composite thin film (Figure 3d). The observed results indicate that the film surface was altered by adjusting the precursor combination with chitosan for electrosynthesis of the films.

**Figure 4:**
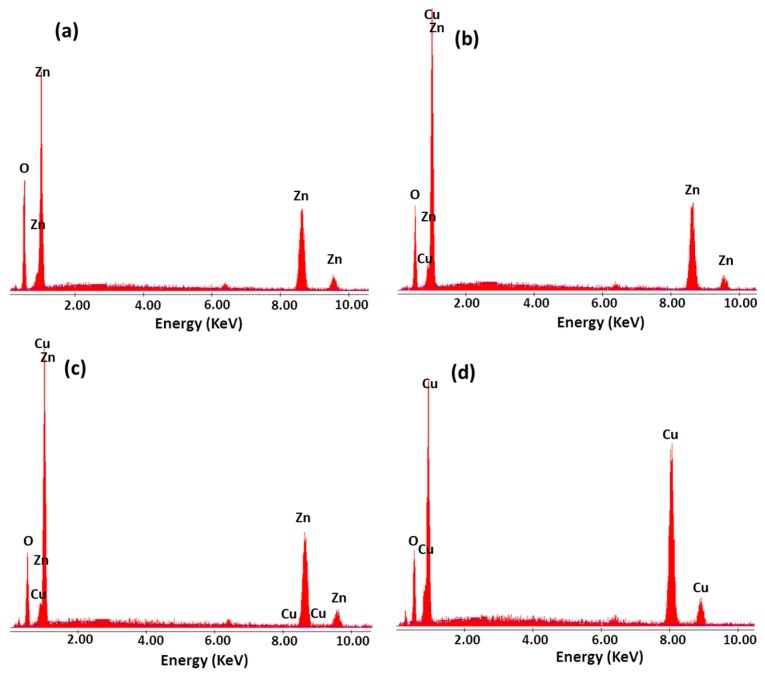
EDAX spectra of electrosynthesized composite thin films: (**a**) chitosan-ZnO; (**b**) chitosan-Zn_0.6_Cu_0.4_O; (**c**) chitosan-Zn_0.3_Cu_0.7_O; and (**d**) chitosan-CuO.

The nanostructure formation with stoichiometric composition were confirmed by energy dispersive analysis by X-rays (EDAX) studies [57]. The composition ratio of the metal oxides combined with chitosan in the composite thin films using EDAX are shown in Figure 4a–d. Chitosan-ZnO composite thin film had a combination mixture of 32.80 and 36.66 corresponding to Zn and O, respectively, as shown in Figure 4a. The EDAX spectrum of chitosan-Zn_0.6_Cu_0.4_O composite thin film (Figure 4b) revealed the atomic percentage of Zn, Cu, and O to be 22.58, 14.72, and 31.88, respectively. From Figure 4c, the observed atomic percentages of Cu, Zn and O were 26.80, 12.46, and 33.66, respectively, for the chitosan-Zn_0.7_Cu_0.3_O composite thin film. The compositional ratio of Cu and O were 29.97 and 34.91, respectively, in the chitosan-CuO thin film (Figure 4d). 

**Figure 5:**
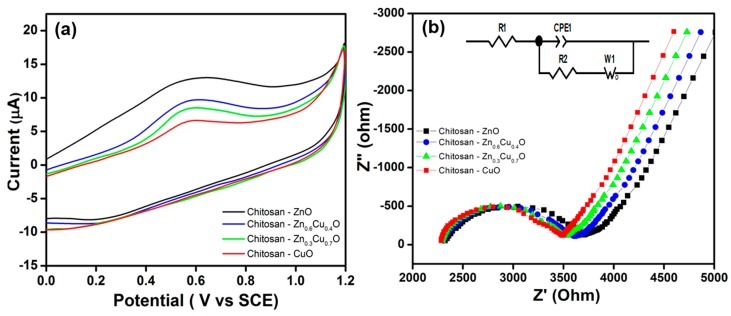
(**a**) CV curves and (**b**) impedance spectra for the electrosynthesized composite thin films. Inset—the Randomize-Simplex model fitted circuit consisting of resistor R_1_ in series with a parallel combination of R_2_ C_2_, which is in series with W_3_.

Figure 5a shows the cyclic voltammograms (CVs) of the electrosynthesized composite thin films recorded using the composite thin films as electrodes with an electrolyte solution of 0.1 M HCl at a scan rate of 20 mV s^−1^. The electrosynthesized composite thin films showed good redox electrochemical behavior in acidic solution with an anodic peak obtained at around 0.18 V vs. a saturated calomel electrode (SCE), while a cathodic peak observed at around 0.55 V vs. SCE for chitosan-ZnO. In addition, the cathodic peak shifted toward negative and the anodic reaction rate decreased with a decrease in Zn content in the chitosan-Zn_1−x_Cu_x_O composite films. The positive shift of chitosan-ZnO thin films depressed the anodic current as they offered greater resistance [58]. It is evident that the electrosynthesized composite thin films obeyed the expected electron transfer in an acidic medium [59]. In this case, the chitosan-ZnO electrosynthesized thin films acted as effective conductivity barriers [60]. 

Electrochemical impedance spectroscopy (EIS) analysis was carried for the electrosynthesized chitosan-Zn_1−x_Cu_x_O composite thin films at room temperature, as shown in Figure 5b. The semi-circle part of the curve shortened with an increase in Cu atomic percentage in the chitosan-Zn_1−x_Cu_x_O film [61]. High resistivity was observed for the chitosan–ZnO thin film, which might have been due to more defects caused by the larger grain size [62]. The semicircle was attributed to the grain boundary and the straight line indicated electron transport at the thin electrode/electrolyte interface. The resistance decreased obviously for chitosan-Zn_0.6_Cu_0.4_O, chitosan-Zn_0.3_Cu_0.7_O, and chitosan-CuO composite thin films compared to chitosan-ZnO due to the grain boundary effect. The estimated EIS parameter values are given in Table 1. We can see that the bulk resistance values decreased with respect to the percentage of Cu atoms incorporated into the chitosan–ZnO matrix, which increased the conductivity of the chitosan-Zn_1−x_Cu_x_O (x = 0.4, 0.7, and 1) matrix system. Similarly, Lee et al. [63] observed a decrease in resistance with an increase in Sn cation composition in ZnO. 

**Figure 6:**
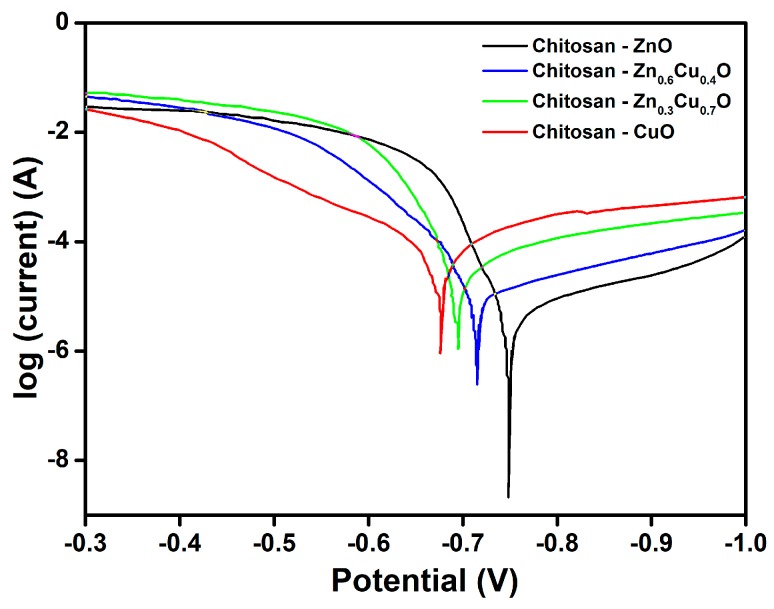
Tafel polarization curves for electrosynthesized composite thin films.

Anodic corrosion was recorded for the electrochemical route prepared for chitosan-ZnO, chitosan-Zn_0.6_Cu_0.4_O, chitosan-Zn_0.3_Cu_0.7_O and chitosan-CuO composite thin films on steel electrodes in 3% NaCl (*w*/*v*) medium. The coated steel surface was maintained under potentiodynamic conditions with a potential sweep between −0.5 and −0.6 V vs. SCE. The corrosion current densities (*j*_corr_) were obtained by extrapolating the linear portions to zero in Tafel plots. Similar observations were reported for copper oxides with a corrosion mechanism by Wan et al. [64]. The corrosion current and corrosion potential were determined by extrapolating the linear portions of the anodic and cathodic Tafel curves from Figure 6, which clearly show the corrosion current density and potential of the various chitosan-based composites. The corrosion current density of the chitosan-ZnO thin film coated steel electrode was 2.81 × 10^−6^ A/cm^2^, which was quite low compared to the values for the other composites. The polarization curves determined that the electrochemically synthesized chitosan-ZnO coating inhibited the anodic dissolution of steel in the corrosive solution. The corrosion rate was evaluated in accordance with the following equation [65]:

*CR* = 3272(*j*_corr_*EM*))/*Ad*, (2)

where *CR* is the corrosion rate in mm per year, *j*_corr_ is the corrosion current density in cm^−2^, *EM* is the equivalent molar mass of the oxidized element in g/equiv molar mass, *A* is the surface area of the specimen in cm^2^ and *d* is the density of the specimen in g/cm^3^. 

The corrosion rate of chitosan-ZnO was 34 × 10^−3^ mm/y due to its low current density of 2.81 × 10^−6^ A/cm^2^, indicating that it had higher corrosion resistance compared to the other composites. The chitosan-Zn_0.6_Cu_0.4_O composite thin film’s corrosion resistance value was 99 × 10^−3^ mm/y with a slightly bowed polarization curve and its current density was 8.12 × 10^−6^ A/cm^2^, as shown in Figure 6. Furthermore, the chitosan-Zn_0.3_Cu_0.7_O composite thin film corrosion resistance value was found to be 320 × 10^−3^ mm/y with a current density of 2.63 × 10^−5^ A/cm^2^. Finally, the chitosan-CuO composite thin film corrosion resistance value was 623 × 10^−3^ mm/y with a semicircle of the polarization creating a semicircle on the plot, and its current density value was estimated at 5.12 × 10^−5^ A/cm^2^. From the above results, we can confirm that the chitosan-ZnO composite thin film exhibited the best corrosion resistance. 

For comparison purposes, the corrosion rate (*CR*) estimated using the ASTM standard weight loss method [66,67] with the following equation:

*CR* = *KTdW*/*A*, (3)

where *CR* is corrosion rate in mm/year (mmpy), *K* is a unit conversion constant, *T* is the period of immersion in hours, *A* is area of the specimen, *W* is weight loss in grams, and *d* is the metal density in g/cm^3^. 3 M NaCl was used as a corroding reagent. Before the analysis, the different composites coated films were weighed precisely to an accuracy of three decimal places. The specimens were immersed in the corrosive environment for 5 h, after which the corroded composite thin films were removed from the corroding reagent and then washed with distilled water. The weight of the corroded composite thin films was measured to an accuracy of three decimal places. The weight loss results presented in Table 1 revealed similar behavior of electrochemical corrosion pattern to the first corrosion rate experiment. 

A schematic representation of the corroded surface of a composite film onto a steel substrate is presented in Figure 1c. The corroded structures created by the corrosion process were due to the flow of current from the anode to the cathode through ionic conductivity and from cathode to anode by the chitosan complex structure through electric conductivity [68,69]. Zn metal oxidation occurred at the anode whereas the chitosan complex hydrogen or oxygen reduction occurred at the cathode, which stimulated localized corrosion over the surface [69]. The following mechanism was derived from the observed trend of corrosion behavior of the chitosan-Zn_1−x_Cu_x_O composite thin films: strong oxide bonding formation between Zn and chitosan; stable surface structure of the nano-slab-like morphology; and higher thickness, lower conductivity, and higher bulk resistance of the chitosan–ZnO complex compared to the other composites [48,69]. 

## 3. Materials and Methods

The electrochemical preparation route used to synthesize the chitosan-Zn_1−x_Cu_x_O composite thin films by galvanostatic mode. In this work, we used 90% deacetylated chitosan (molecular weight 90 Da) for preparation of the composite thin films. The other precursors used to prepare the electrolytic bath solutions were ZnCl_2_, CuCl_2_, and acetic acid. In this film deposition method, mild steel, zinc, and SCE were used as the working, counter, and reference electrodes, respectively. 

The cathodic substrate was etched by polishing mechanically to obtain a smooth surface, degreased with trichloroethylene and acid to remove impurities, and then cleaned using de-ionized water and acetone solvent. The anodic material was cleaned using nitric acid solution and acetone. The solution bath was adjusted from pH 1–3 using an acid, the deposition current value was fixed at 2 mA/cm^2^, and the deposition time was fixed at 30 min. All the compounds were mixed as a homogeneous solution in a 100 mL beaker using a mechanical shaker.

Chitosan (0.8 g) was dissolved in 1% acetic acid solution and then used as a bath precursor for electrosynthesis. For chitosan-ZnO nanocomposite preparation, 0.5 M ZnCl_2_ solution was mixed with as-prepared chitosan solution in an electrolytic bath. The chitosan-Zn composite thin film was deposited using the aforementioned electrodeposition parameter values. For the chitosan-CuO composite, 0.5 M CuCl_2_ solution was combined with as-prepared chitosan solution in an electrolytic bath and prepared using the same deposition parameters. Using these and the same as-prepared chitosan solution, the electroplated chitosan-Zn_0.6_Cu_0.4_O composite film was prepared with 0.5 M ZnCl_2_ and 0.2 M CuCl_2_, and the chitosan-Zn_0.3_Cu_0.7_O composite film was made using 0.1 M ZnCl_2_ and 0.3 M CuCl_2_.

The prepared composite thin films were characterized using the following instruments to analyze their properties. The structural properties of prepared composite thin films identified using an X-ray diffractometer (X’Pert PRO PANalytical diffractometer) with Cu K_α_ radiation (λ = 0.15406 nm) and a scanning rate of 0.01°/step in the 2θ range of 10° to 80°. The surface morphology and compositional analysis of the composite films carried out using EDAX attached to a scanning electron microscope (Hitachi-S3000, Tokyo, Japan) to determine their size, shape, and composition. A three-electrode cell system consisting of a nanocomposite-coated mild steel specimen as a working electrode, SCE as a reference electrode, and platinum (Pt) as a counter electrode was used for the electrochemical measurements. The corrosion behavior of nanocomposite-coated mild steel specimens were evaluated in 3.0% NaCl solution with a potential sweep rate between −0.5 and −0.6 V vs. SCE. For comparison purposes, corrosion testing was performed using the ASTM weight loss method at room temperature (~27 °C). The impedance analysis was carried out using an Autolab BSTR 10A instrument (Metrohm Autolab B.V., Utrecht, The Netherlands). AC signal with amplitude of 50 mV and frequency range from 0.05 to 10^5^ HZ were used to study the performance of the thin films. CV measurements were carried out in 1M HCl using a CHI 1022 electrochemical analyzer/workstation (CH Instruments, Bee Cave, TX, USA).

## 4. Conclusions

Composite thin films of chitosan-ZnO, chitosan-Zn_0.6_Cu_0.4_O, chitosan-Zn_0.3_Cu_0.7_O, and chitosan-CuO were prepared by electrosynthesis in galvanostatic mode. The prepared composite thin films’ structural, morphological, compositional, corrosion resistance, and impedance properties were plausibly studied using XRD, SEM, EDAX, Tafel polarization, and impedance spectroscopy, respectively. Dual-phase nature was observed for the chitosan-Zn_1−x_Cu_x_O composite thin films. The morphological properties of the chitosan-Zn_1−x_Cu_x_O thin films were enormously varied by precursor concentration in an electrolytic bath. Nano slabs and spherical-shaped grains were observed in the SEM micrographs of the composites. EDAX spectra revealed the atomic percentage values of electrosynthesized chitosan composite thin films. The best corrosion resistance performance was evident in the chitosan-ZnO thin film compared with other composite thin films, and it could be a potential material for applications requiring corrosion resistance.

## Figures and Tables

**Table 1:** EIS and corrosion parameters of electrosynthesized composite thin films.

Composite	EIS Parameters			Corrosion Parameters			
				Electrochemical Method		Weight Loss Method	
	R1 (Ohm)	R2 (Ohm)	CPE1 μF	Corrosion Current Density (cm^−2^)	Corrosion Rate (mmpy) 10^−3^	Weight Loss (mg)	Corrosion Rate (mmpy) 10^−3^
Chitosan-ZnO	2321	3699	0.63	2.81 × 10^−6^	34	160	35
Chitosan-Zn_0.6_Cu_0.4_O	2308	3610	0.58	8.12 × 10^−6^	99	260	58
Chitosan-Zn_0.3_Cu_0.7_O	2290	3510	0.52	2.63 × 10^−5^	320	415	93
Chitosan-CuO	2282	3488	0.31	5.12 × 10^−5^	623	635	141
